# Designing a framework for curriculum building in systematic review competencies for librarians: a case report

**DOI:** 10.5195/jmla.2024.1930

**Published:** 2024-10-01

**Authors:** David P. Farris, Rachael A. Lebo, Carrie Price

**Affiliations:** 1 dpfarris@mdanderson.org, Research Services Librarian, Research Medical Library, MD Anderson Cancer Center, Houston, TX; 2 Rachael.lebo@usd.edu, Clinical Services Librarian, Wegner Health Sciences Library, University of South Dakota, Sioux Falls, SD; 3 carrie.price@nih.gov, Biomedical Librarian, NIH Library, Office of Research Services, OD, National Institutes of Health, Bethesda, MD

**Keywords:** Teaching, curriculum, systematic reviews, expert searching

## Abstract

**Background:**

Librarians play an important role on systematic review teams because of their expertise in information organization, database searching, and records management. Many systematic review training opportunities exist, but not all are tailored to the needs of librarians. The Medical Library Association, along with a workgroup of experts on systematic reviews and review services, developed a Systematic Review Services Specialization (SRSS) that was launched in 2022. One of the required courses in the specialization was developed by the authors, who set out to build a value-added curriculum that would provide essential searching skills for librarians working in evidence synthesis domains.

**Case Presentation:**

The authors present a case report on creating a framework for developing a new course in the Medical Library Association's Systematic Review Services Specialization. The objectives of the course were intended to align with six systematic review competencies for librarians developed and published by a group of health science librarians from the University of Michigan in 2017, which include 1) conducting a reference interview, 2) performing preliminary searches, 3) selecting appropriate resources to search, 4) building an extensive, comprehensive, and documented search strategy, 5) peer reviewing search strategies, and 6) reporting search methods. With these objectives in mind, the instructors created four separate modules and an activity.

**Conclusion:**

Systematic review skills for librarians are essential to many health science library jobs since librarians are considered important collaborators within systematic review teams. Through eleven cohorts of the course held over 2022–2024, the authors constructed and delivered a comprehensive curriculum.

## BACKGROUND

Health science librarians are becoming essential members of systematic review teams. Research has shown that librarian co-authors were positively correlated with higher-quality reported search strategies [1–5]. Other investigations have demonstrated that a librarian plays a central role on a systematic review team beyond searching for literature [6–9]. Most methodological guidance, including the *Cochrane Handbook for Systematic Reviews of Interventions, JBI Manual for Evidence Synthesis, Campbell Collaboration*, and the *National Academies for Science, Engineering, and Medicine*, states that an information retrieval expert should be consulted for the literature search [10–13]. The *Cochrane Handbook* notes that “review authors should work closely, from the start of the protocol, with an experienced medical/healthcare librarian or information specialist“ [14].

Comprehensive searches “aim to be as extensive as possible in order to ensure that relevant studies as possible are included in the review” [14]. Formal literature searches are often complex, containing multiple concepts that require knowledge of individual database syntax and Boolean operators. Systematic searches aim to avoid selection or information bias that could occur with the use of inappropriate search terms, the omission of related terms, or the failure to apply an objective and reproducible search to a “range of sources” [15]. Further, the literature searches must be exhaustively documented to ensure transparency and reproducibility.

Many health science librarians work in settings where the provision of systematic review support is growing. In addition to building and documenting comprehensive literature searches, librarians can often find themselves providing advice on methods, resources, records management, screening, citations, and more [6]. In 2017, Townsend et al. developed a competency framework for librarians involved on systematic reviews [16]. This framework, adapted from Miller’s Pyramid for Clinical Assessment, outlines six competency areas with smaller associated skills and indicators [17]. The Medical Library Association's (MLA) Systematic Review Services Specialization (SRSS) Systematic Review (SR) Workgroup, composed of systematic review experts and later, Systematic Review Caucus members, used the Townsend et al. competencies to define twenty-six fundamental skills on two Learning Pathways (Level I and Level II), which are satisfied through various courses, both elective and required, within the SRSS [16, 18, 19]. For a list of the twenty-six fundamental skills, see Appendix A in the Supplementary Material list.

While other systematic review training opportunities exist, some 1) are not tailored to the needs of librarians or do not include librarians on the instructor team, 2) have been discontinued, or 3) have access barriers. Librarians teaching librarians, as described in this case report, is pivotal for providing opportunities of peer knowledge sharing, discussing best practices in searching, empowering librarians as researchers and collaborators, and developing systematic review services to meet user needs. The MLA's SRSS courses, and the course described in this case report, can be a robust complement to a librarian's systematic review training.

## CASE PRESENTATION

In this case report, the authors describe their approach to establishing a thorough and engaging curriculum for the required SRSS Level I core course, *Essential Searching Skills for Librarians on Systematic Review Teams*, with the goals of meeting the objectives established by the SR Workgroup of the MLA and to build confidence in participants embarking on systematic review support [18]. Individuals who wish to earn the Systematic Review Services Specialization must take eight required core courses and four electives selected from a course list. The pilot class was held in person at the MLA Annual Meeting in New Orleans in May 2022. Subsequently, the course was offered online in October 2022; February, March, April, May, August, October, and December 2023; March 2024, and once again in person in May 2024 at the Annual Meeting in Portland, Oregon. Each class had approximately thirty registered participants who were added to a Slack channel for the purposes of course information dissemination and communication. The instructors established an open course site on Open Science Framework (OSF), with links to required and supplemental readings, digital handouts for various topics, slides, and instructions for the course activity.

### Course Planning and Structure

Through a series of virtual meetings and emails spread out over several months leading up to the pilot course, the instructors discussed the course objectives and how best to deliver and achieve them. Four modules were established that would meet the course objectives: Module 1, consultation and pre-searching, Module 2, searching, Module 3, search strategy peer review, and Module 4, search reporting. Table 1 outlines the learning objectives provided to the instructors by the MLA SR Workgroup, the curriculum module in which those objectives are met and their corresponding SRSS skills.

**Table 1:** Learning Objectives for Essential Searching Skills for Librarians on Systematic Review Teams

Learning Objective	Covered In	Maps To SRSS Skill
Determine the need for a systematic review.	Module 1	8
Conduct preliminary searches.	Module 1	8, 9
Select appropriate databases.	Module 2	9
Build a comprehensive search strategy.	Module 2	10, 13
Review search strategies.	Module 3	11
Report search methods.	Module 4	12, 14

Because the course is a requirement in the SRSS Level I Learning Pathway, the content is intentionally designed for searchers of all levels, since both new and experienced searchers earning the specialization enroll. PubMed is the primary platform used for the class, although participants are encouraged to begin their searches with the database and platform they feel most comfortable using when doing their own searches. PubMed is chosen because it is fully accessible without restriction to anyone with internet access and incorporates the MeSH database of controlled vocabulary, which is relied on heavily for terminology harvesting and knowledge-building.

The pilot in-person offering was allotted a total of four hours, while the online offerings have been divided into two 2.5-hour sessions. The following elements of the case presentation focus on the virtual offerings, since they have been in the majority, and are not meant to present the detailed and proprietary content of the class, but rather to provide a useful framework to others who may wish to establish or investigate building a curriculum for the purposes of enhancing systematic review searching.

### The Course

#### Introduction to the Material

During the introduction to the course, participants are asked to take a five-question pre-test. The instructors introduce themselves and provide logistics for the course. Prior to beginning Module 1, the instructors also discuss persistently perplexing terminology within the field, such as distinguishing between databases and platforms, describing natural language search terms, and identifying the myriad ways in which database providers name their thesauri and controlled vocabulary. The instructors then describe the schedule for what will be covered and in what order. Transitions are accommodated with a pause for questions and discussion before moving on. Use of chat, live discussion, and other modalities of interaction are strongly encouraged to support participant engagement.

#### Module 1: Consultation and Pre-Searching

The first module focuses on doing a cursory search of the literature and conducting the reference interview. The instructor emphasizes the importance of librarians understanding the impact of systematic review collaborations on their time and workloads, as projects like these can often take more work than initially expected.

The module starts with a discussion about doing a preliminary search on the research topic, including searching for systematic reviews already published, and how to find systematic reviews that are in-progress. Finding non-systematic review articles is also covered to illustrate ways to identify relevant keywords and spelling variations in order to begin compiling a list of terms that can be used in the search strategy. In addition to keywords, systematic reviews and other articles identified are also used to start gathering a list of possible controlled vocabulary terms.

The second part of Module 1 focuses on the specialized reference interview, asking questions, and setting expectations. For the initial reference interview, the benefits of asking open-ended questions and conducting in-person or virtual meetings rather than relying only on e-mail exchanges are stressed. The reference interview should be used to ask more in-depth questions about the research topic, inquire if anyone on the research team previously worked on a systematic review, discuss key aspects of the methodology and finally, ask about restrictions to the search. A variety of restrictions, such as age group, language, and geographic location, initiate instructor and participant debates about how and whether restrictions can be applied without introducing bias. The topics of database selection, clinical trials, grey literature, hand-searching, preprints, and a brief introduction to drug and chemical searching are also covered during this section.

#### Module 2: The Search

Module 2 contains the most content. Instructors present their individual methods for search documentation and introduce five question development frameworks, such as the well-known PICO (population, intervention, comparison, outcome) and several others, for use as an aid in identifying distinct search concepts. This is followed by an overview of concept nesting, which entails building smaller search concepts to be combined into a larger search.

The next section includes a review of database controlled vocabulary, which are used to locate subject headings and identify information in the record to determine the heading's relevance to the topic. Examples from the MeSH and Emtree thesauri are used to demonstrate these topics. Although MeSH terms are openly available through PubMed, Emtree terms are available only through licensed resources, so not everyone will have access. Emtree terms are demonstrated, however, since the Emtree thesaurus provides important information for searchers and should be used if access is available. The next section delves into advanced searching techniques, syntax, and database documentation. Differences among various databases and platforms are highlighted, and a discussion of filters and hedges completes the lesson.

A live demonstration of building a thorough and welldocumented search in PubMed using the techniques discussed in class, such as term-harvesting and controlled vocabulary, is followed by an introduction to the course activity. The course activity involves constructing a search string for one of three concepts that are part of a larger research question. Participants are given time to discuss their approaches in smaller groups. This abbreviated activity is chosen over a full systematic review search to accommodate the participants in skill-building. The time constraints and the participants' various levels of database searching skills are factors in choosing this modality. Building a full systematic review search can take several weeks, requires input from an actual research team, and is not feasible in the context of this course. Participants are dismissed from the first virtual session at this point, returning in the second virtual session where they assemble once again in their smaller groups to discuss, finalize, and submit their search concepts. After the breakout groups, the instructors live-test the full threeconcept search strategy in PubMed, identify potential key articles, and share tools that could help expedite translating the search to other databases. There is time for discussion regarding what questions would have been asked of a real, not theoretical, research team. Some additional searching skills, like testing for errors, proofreading, ensuring the inclusion of key articles, and translating equivalently across platforms and databases are covered.

Finally, the instructors examine both open and subscription drug information resources in more depth. One drug is used as an example to demonstrate differences in generic, brand, alternative, and chemical names, all of which may be used in systematic searching.

#### Module 3: Peer Reviewing a Search with Peer Review of Electronic Search Strategies (PRESS)

The third module presents a comprehensive review of the Peer Review of Electronic Search Strategies (PRESS) 2015 Guideline Statement [20], which provides an evidence-based guideline for peer review. The instructor discusses the rationale for search peer review. Several tools are demonstrated to assist librarians with the process such as the PRESSforum website, which is a repository for tutorials, files, and videos that illustrate the entire process and a place where librarians can request and offer peer review of their search strategies [21]. The PRESSforum website also provides a link to the latest version of the PRESS Search Submission and Peer Review Assessment form, which is available via the CADTH website as a Word file and interactive PDF [22]. The module concludes with a demonstration and brief discussion of various suggestions that can be implemented to evaluate each element of a search strategy, including question translation; Boolean and proximity operators; subject headings; text word searching; spelling, syntax, and line numbers; and finally, limits and filters.

#### Module 4: Reporting the Search with PRISMA-Search

Module 4 covers the Preferred Reporting Items for Systematic Reviews and Meta-Analysis Search (PRISMA-S) extension and gives an overview of best practices in search reporting [23, 24]. Sixteen items are described, with examples of reporting for each, which are categorized into four parts according to the publication: 1) information sources and methods, 2) search strategies, 3) peer review, and 4) managing records. The participants then have the opportunity to provide feedback on several selected published search methods using the categorized items from PRISMA-S.

### Conclusion of the Course

Having covered the reference interview and pre-searching, search documentation, syntax, term harvesting, search peer review, and search reporting, the instructors discuss their own anecdotal experiences as librarians on systematic review teams and provide time for an open-ended discussion. Finally, participants are asked to complete a post-test, identical to the pre-test, provided by the instructors, and a course feedback form provided by MLA.

#### Course Feedback and Performance

Comments from the course feedback forms were consistently positive. Recurring themes were centered around 1) the instructors' abilities to curate content for both new and experienced searchers, 2) enthusiasm for the breakout rooms and the course activity, and 3) the ability to incorporate something new from the class into their professional work. Constructive critiques ranged from 1) not enough time to too much time, 2) a desire for individual feedback on an individual search, and 3) frustration with the number of communication tools used during the course (see Appendix B in Supplementary Material: Selected Feedback). For each iteration of the course, the instructors consider previous cohorts' feedback and make small adjustments as necessary. For example, an early cohort disclosed that they did not understand the idea of concept nesting, so concept nesting instruction was provided in greater detail. Other cohorts reported that they enjoyed the time spent in smaller groups and found it valuable, so the instructors lengthened the amount of time that participants spent in small groups to support time for individual discussion. An emerging theme on feedback from all cohorts was the realization that there are no absolute answers in systematic searching. The instructors have not received feedback that necessitated larger changes.

As noted, the course pre-test and the post-test were identical. The five questions cover both confidence level in searching and knowledge items covered specifically within the course. The participant pre-test/post-test scores demonstrate an increase in knowledge for skills important to systematic review searching which are outlined in the list of SRSS skills (Table 2).

**Table 2:** Pre-Test/Post-Test Analysis

Test Question	Unit	Pre-test	Post-test	Difference	SRSS Skill
How confident are you at building a comprehensiv e literature search? (1=least, 5=most)	Mean	3.32	3.96	+0.64	8–14
The Cochrane Handbook Recommends searching which three resources?	Percent Correct	74	84	+10	9
Select appropriate drug resources (participants were asked to select all appropriate resources from a list).	Percent Correct	92	96	+4	9
PRESS Statement contains (participants were asked to select the elements of the PRESS publication)	Percent Correct	62	70	+8	11
PRISMA-Search contains (participants were asked to select the major categories of the PRISMA-S)	Percent Correct	62	78	+16	14

## DISCUSSION

For the instructors, the question of “*Does this add value?*” has been a guiding principle when deciding how to structure the course and when to implement changes. The instructors usually learn something new from the participants, as many of them have been willing to share unique tips and new knowledge about platform vendors and database updates with the group. The bulk of the course content (Modules 1 and 2) focuses on search development techniques such as using both keywords and controlled vocabulary, searching multiple resources, not relying on database filters, implementing field tags, and searching each resource in an equivalent manner, which are important best practices for systematic searching. The instructors continue to believe that one of the most valuable takeaways of the course is that the searcher has a great deal of autonomy in deciding how to develop, build, and document the search regarding the order and structure of the terms and the platforms used for documentation. What matters the most is that the documented searches are sensitive in nature and easily reproducible.

Librarians who support systematic reviews and other evidence syntheses can benefit from the opportunity to engage in the course *Essential Searching Skills for Librarians on Systematic Review Teams*. Participants have a chance to meet and chat with other librarians supporting similar services and doing comparable work, observe searching processes other than their own, identify existing guidance documentation, gain practice in building a comprehensive concept, and determine best practices for establishing future systematic review collaborations and instruction. Librarians looking to develop systematic review instruction may find this case report beneficial in instituting their own instructional materials and/or curriculum. For these librarians, it may also be a worthwhile task to review the systematic review competencies devised by Townsend et al., as well as the roles for librarians on systematic review teams described by Spencer and Eldredge, as the instructors of this course did when initially planning and developing the curriculum [6, 16].

As evidence synthesis methodologies continue to evolve, author teams cannot overlook the profound impact of librarians and their search expertise in supporting evidence synthesis methodologies. The development of proficient searching skills and searching instruction techniques among librarians is essential for mitigating bias, ensuring credibility, and upholding the integrity of published evidence syntheses. This case report demonstrates how the instructors, through diligent planning and subject matter expertise, built a robust course curriculum, thereby playing a crucial role in supporting the professional development of librarians supporting systematic review services and earning their Systematic Review Services Specialization.

## Data Availability

Data associated with this article are presented in Appendix A (SRSS Skills) and Appendix B (Selected Feedback).

## SUPPLEMENTAL FILES

**Appendix A:** Systematic Review Services Specialization: SRSS Competencies

**Appendix B:** Selected Participant Feedback
