# Supplementary material for: Designing a framework for curriculum building in systematic review competencies for librarians: a case report
Source: J Med Libr Assoc. 2024 Oct 7;112(4):357–63. doi: 10.5195/jmla.2024.1930 (PMC11486078; doi:10.5195/jmla.2024.1930)
Supplement: Supplementary file 1 — Appendix A: Systematic Review Services Specialization: SRSS Competencies [file jmla-112-4-357-s01.docx]

# The Systematic Review Services Competencies

From: Professional Development: Systematic Review Services Specialization. MLA. Accessed 29 April 2024. <https://www.mlanet.org/page/systematic-review-services-specialization>

# What Skills Does the SRSS Address?

The SRSS is built upon the Systematic Review Services Competency:

Health and biomedical information professionals with competency in Systematic Reviews use a range of information retrieval and management knowledge and skills to support users and researchers. They promote the use of established guidelines, best practices, ethical synthesis practices, and transparent reporting in the service of high quality, reproducible scientific and biomedical research.

The Systematic Review Competency has two levels, Level I and Level II. Each level has skill indicators organized by topic.

# Level I

Your Level I SRSS certificate will give you a solid grounding in the fundamentals of systematic reviews and prepare you to assist clinicians and researchers in conducting health sciences-related systematic reviews. With this accomplishment, you will not only have systematic review skills, you will be able to offer systematic review services with increased confidence and ease, expanding your career and job possibilities.

See the [Systematic Review Services Level I Course List](https://www.mlanet.org/page/srsscourses) for the core and elective courses.

## Fundamentals of systematic reviews

1. Explain the major steps in the design and conduct of a systematic review
2. Explain how systematic reviews differ from narrative reviews
3. Explain how systematic reviews inform practice, policy, and research
4. Explain the value of established systematic review guidelines and standards

## Communication

5. Negotiate involvement in systematic review projects 
6. Negotiate recognition of librarian's intellectual contribution to a systematic review
7. Collaborate with researchers to implement effective search strategies

## Searching

8. Conduct searches to confirm the need for a systematic review
9. Select databases and other resources appropriate to a research question
10.  Develop an extensive, effective, systematic, and replicable search strategy for each resource appropriate to a research question
11. Review search strategies under development using the PRESS (Peer Review of Electronic Search Strategies) rubric

## Documentation & Reporting

12. Describe planned search methods
13. Document search processes, including search iterations and decision-making
14. Report search methods for publication in conformance with established reporting standards

## Data Management I

15. Utilize citation management software
16. Inform researchers about available systematic review software

# Level II

Your Level II SRSS certificate will enable you to serve as an expert resource for systematic review research teams on a wide range of systematic review tasks. You’ll also be able to develop or enhance the systematic review service at your library, serve as a peer reviewer for systematic review articles, and critically appraise published systematic reviews. Earning your Level II certificate will boost your skills and expertise, enhance your value as a partner in evidence synthesis research, and expand your career possibilities!

See the [Systematic Review Services Level II Course List](https://www.mlanet.org/page/systematic-review-services-specialization-level-ii-course-list) for the core and elective courses.

## Project Planning

17. Match a research question to an appropriate evidence synthesis type
18. Advise systematic review teams on timelines, team composition, and productivity tools
19. Collaborate with systematic review teams on developing systematic review protocols

## Quality & Reproducibility

20. Advise teams on selection and use of data extraction tools
21. Advise teams on selection and use of risk of bias tools
22. Prepare search-related data for deposit or archiving

## Searching

23. Identify and use search filters

## Peer Review & Appraisal

24. Peer review manuscripts for publication
25. Appraise the quality of published systematic reviews

## Service Management

26. Implement best practices for establishing, managing, and evaluating systematic review services
