# Supplementary material for: Designing a framework for curriculum building in systematic review competencies for librarians: a case report
Source: J Med Libr Assoc. 2024 Oct 7;112(4):357–63. doi: 10.5195/jmla.2024.1930 (PMC11486078; doi:10.5195/jmla.2024.1930)
Supplement: Supplementary file 2 — Appendix B: Selected Participant Feedback [file jmla-112-4-357-s02.docx]

# Positive Feedback

• I think the exploration of the complex approach to building a search was very beneficial. The fact that there is no one right way to build a search I always feel I am missing out on something to make a search stronger. This course helped me to build my skills further.

• I think the creation of the search strategy and talking about it with colleagues was really helpful. I think it also allows everyone to show that searches can be done differently, but we do have an over-branching guidance on what librarians should follow for systematic review searching. I think it was a great idea to split up the content into two different sessions.

• I am part of a systematic review service at my workplace. I am happy to have these learning opportunities to make my job easier.

• The facilitators did a great job of accommodating a WIDE variety of skillsets across the participants and made the coursework inclusive and engaging, even for more seasoned experts.

• Nice mix of basics with well thought-out best practices. As an experienced searcher, was a bit concerned I wouldn't gain much and was pleasantly surprised.

• The instructors were amazing! I learned new things as well as had previously learned things reinforced. And they made it such a comfortable and pleasant space for learning.

• This class was hands down the best class in the whole SR process. There was practical useful information, well there were in all the classes, but this one allowed us to put the knowledge to use almost immediately. I did have problems using the Slack app, but that was due to operator error on my end. This class could have easily been a weeklong but the team did a phenomenal job of keeping things on point and in order and with examples and keeping things interactive. Well done! I look forward to seeing more webinars and trainings offered by these three.

# Areas for Improvement

• I expected a very interactive course, and the presenters definitely delivered. My only comment was maybe there were too many tools for making it interactive (Slack, course wiki, Padlet, Slido, etc.). It created a lot of tabs for someone who already has lots of tabs open usually (I am sure I am not the only librarian with the too many tabs problem!) and it was a little hard to keep track.

• I would have loved for the other participants in my group to have participated in the Slack group discussion as I was hoping to learn more from my peers.

• While I might suggest a number of other topics that would be good to have as a follow-up - I know that MLA is working on a series of courses that are offered for the Systematic Review Services Specialization. So I think as long as those courses continue to be updated or added to the specialization track, no follow up would be needed. I guess maybe you could offer a course where individuals could bring a systematic review search to the course and another person in the class could peer review it.

• Unfortunately, with the large amount of information to be covered, the presenters had to move quickly through the material. In addition, I am a very slow typist, so by the time I was able to type a response and/or question in the chat box, the class had progressed beyond the topic into the next topic. Combining these two challenges, I wasn't able to ask my questions or make my comments quickly enough not to hold the class up retracing a topic that was just finished.

• It would be great if we could have a mini assignment that we could submit for feedback - develop our own search if we have a topic or work on one provided by instructors if not. I think this course could be expanded over 3 or 4 sessions and really get into the iterative search process rather than just a couple demos.

• A simple course website rather than Slack & [OSF], which may be convenient for course owner, but just one more unfamiliar community to navigate for some participants.

• I would have liked them to include resources for additional community support like Listservs and other discussion forums for SRs. Just a list on a slide would be sufficient for future reference. You didn't really talk a lot about registering a SR protocol. Touched on it a little but more would be good.
